# Supplementary material for: Wildlife Photos on Social Media: A Quantitative Content Analysis of Conservation Organisations’ Instagram Images
Source: Animals (Basel). 2022 Jul 12;12(14):1787. doi: 10.3390/ani12141787 (PMC9311588; doi:10.3390/ani12141787)
Supplement: Supplementary file 1 [file animals-12-01787-s001.zip › Table S1.pdf]

Table S1. Count and percentage of image elements for the final sample (n = 670). Counts may not all be equal due to missing values. Animal Features and Human Features are counted by individual in the image.

**Image Characteristics**			**Animal Features**			**Human Features**		
	**Count**	%		**Count**	%		**Count**	%
**Is there text on the image?**			**Taxon of the animal**			**Sex of the human**		
Yes	116	17.34	Invertebrate	70	7.87	Male	41	27.15
No	553	82.66	Mollusc	11	1.24	Female	45	29.80
**What type of image is it?**			Fish	34	3.82	Undetermined	61	40.40
Photo	663	99.1	Bird	261	29.36	**Age of the human**		
Cartoon	1	0.15	Mammal	394	44.32	Infant	2	1.32
Illustration	4	0.6	Amphibian	32	3.6	Child	14	9.27
Computer Drawing	1	0.15	Reptile	87	9.79	Teen	3	1.99
**What is the image's colour palette?**			**Type of shot of the animal**			Adult	84	55.63
Full Colour	651	97.31	Full body	540	61.22	Elderly	2	1.32
Black and White	12	1.79	Half body	254	28.8	Undetermined	46	30.46
Obvious Filter	6	0.9	Face	85	9.64	**Role of the human**		
**How is the image quality/resolution?**			Close Up	3	0.34	Celebrity	3	1.99
High	344	51.81	**Where is the animal facing?**			General Public	24	15.89
Medium	242	36.45	Facing Camera	279	31.35	Other	3	1.99
Low	41	6.17	Back to Camera	84	9.44	Undetermined	51	33.77
Poor	37	5.57	Side On	397	44.61	Politician	1	0.66
**What style is the image?**			Face Not Visible	18	2.02	Researcher	7	4.64
Animal Portrait	622	92.84	Looking at Camera	112	12.58	Vet / Nurse	14	9.27
Landscape	20	2.99	**Is the animal alive or dead?**			Wildlife Carer	16	10.60
Human Selfie	27	4.03	Alive	873	98.98	Zookeeper	19	12.58
Other	1	0.15	Dead	9	1.02	Conservationist	10	6.62
**What is the background of the image?**			**Age of the animal**			Student	2	1.32
Naturalistic	452	67.77	Infant	28	3.15	Wildlife Rescuer	1	0.66

Human Made	171	25.64
Blank	31	4.65
Other	13	1.95
**If the background is man-made, what is it?**		
Home	14	11.57
Tank	9	7.44
Zoo	54	44.63
Vet Clinic	10	8.26
Street	3	2.48
Other	31	25.62
**Are there man-made objects in the image?**		
Yes	137	20.45
No	533	79.55
**Is the image graphic?**		
Yes	11	1.64
No	659	98.36
**Is there a human in the image?**		
Yes	116	17.39
No	551	82.61

Juvenile	116	13.03
Adult	740	83.15
Unknown	6	0.67
**Distance of animal from the camera**		
Distant	13	1.46
Far	80	8.99
Medium	299	33.6
Close	445	50
Very Close	53	5.96
**Is the animal brightly coloured?**		
Yes	236	26.67
No	649	73.33

**Type of shot of the human**		
Full Body	24	16.67
Half Body	56	38.89
Face	12	8.33
Body Part	52	36.11
**Is the human wearing a uniform?**		
Yes	44	29.53
No	105	70.47
**Is the human touching an animal?**		
Yes	100	66.23
No	51	33.77
**Is the human holding an animal?**		
Yes	75	49.67
No	76	50.33
**Is the human feeding an animal?**		
Yes	8	5.44
No	139	94.56
**Distance of the human from the animal**		
Touching	98	67.59
Close ~30cm	24	16.55
Far ~1m	15	10.34
Very Far ~5m+	8	5.52
