# Supplementary material for: Wildlife Photos on Social Media: A Quantitative Content Analysis of Conservation Organisations’ Instagram Images
Source: Animals (Basel). 2022 Jul 12;12(14):1787. doi: 10.3390/ani12141787 (PMC9311588; doi:10.3390/ani12141787)
Supplement: Supplementary file 1 [file animals-12-01787-s001.zip › Table S2.pdf]

Table S2. Mean and Median counts of the number of animals and humans within an image.

Variable	Mean	Median
Number of Animals in the image	1.674	1
Number of Humans in the image	0.241	0
