# Supplementary material for: Wildlife Photos on Social Media: A Quantitative Content Analysis of Conservation Organisations’ Instagram Images
Source: Animals (Basel). 2022 Jul 12;12(14):1787. doi: 10.3390/ani12141787 (PMC9311588; doi:10.3390/ani12141787)
Supplement: Supplementary file 1 [file animals-12-01787-s001.zip › Table S3.pdf]

Table S3. The 10 most represented species in the database by count and percentage of total species (n = 147)

	Count	%
Koala	93	10.48
Kangaroo	55	6.20
Lizard	36	4.06
Wallaby	33	3.72
King Parrot	32	3.61
Frog	32	3.61
Snake	28	3.16
Flying fox	25	2.82
Owl	24	2.71
Fairy Wren	24	2.71
Seagull	22	2.48
Quoll	21	2.37
